# Health and wellbeing in informal caregivers and non-caregivers: a comparative cross-sectional study of the Swedish a general population

**DOI:** 10.1186/s12955-015-0309-2

**Published:** 2015-07-29

**Authors:** Erik Berglund, Per Lytsy, Ragnar Westerling

**Affiliations:** Department of Public Health and Caring Sciences, Uppsala University, Box 564, SE-751 22 Uppsala, Sweden

**Keywords:** Self-rated health, Wellbeing, Informal caregiving, Population health

## Abstract

**Background:**

Informal caregiving by relatives is a great resource for individuals as well as for society, but the caregiving role is associated with health problems for the caregiver. This study aimed to compare caregivers’ self-rated health, number of recent days with poor health and psychological wellbeing with that of non-caregivers in a general Swedish population.

**Methods:**

From 2004 to 2013, 90,845 Swedish people completed a postal questionnaire about their health, number of recent days with poor health during last month, psychological wellbeing and if they were performing caregiving or not. Descriptive statistics, chi-square analysis, ANOVA, logistic regressions and negative binomial regression models were used to investigate associations between being a caregiver or not and health and wellbeing. Negative binomial regression was used to assess the relation between caregiver status and recent days with poor health or functioning.

**Results:**

Eleven percent reported having a caregiving role. Caregivers reported poorer self-rated health compered to non-caregivers, also in adjusted models; odds ratio (OR): 1.07 with a 95 % confidence interval (CI): 1.01-1.13. Caregivers also reported lower psychological wellbeing compared to non-caregivers; OR: 1.22, CI: 1.15-1.30. Caregiving status was associated with more recent days with poor physical health and more recent days with poor mental health.

**Conclusions:**

This study suggests that caregivers have worse perceptions in self-rated health and psychological wellbeing compared with non-caregivers, indicating that the role of caregiver is adversely associated with health. This association also appears in terms of reporting days of poor health in the last month. The underlying mechanism of these associations, including the potential detrimental health effects of being a caregiver, needs to be investigated in longitudinal studies.

## Introduction

Informal caregiving entails an individual, usually family member or relative, providing unpaid assistance with a variety of everyday activities to a care receiver. People performing informal caregiving to relatives or others in their surroundings sustain a great resource for the care recipient, the health care system and for society. Informal caregiving lies outside the market economy and is often socially and politically invisible, its economic value is generally not acknowledged but estimated to be of high value [1]. Caregiving is also associated with a burden and may have a deleterious effect on the caregiver’s health-related quality of life leading to an increase in his/her own need of health care [2]. There is strong consensus that caring for individuals with disability or an older family member is burdensome and sometimes becomes stressful to caregiving family members and, thus, may contribute to poorer perceived and mental health [3–6]. Caregiving is also seen as a risk factor for mortality [7]. Research findings indicate that the combination of the physical demands of caregiving, prolonged distress and biological vulnerability of caregivers may compromise their physiological functioning and increase their risk for physical health problems that may lead to increased mortality [7]. Studies have shown high risks for adverse physical and mental health as well as institutionalization and premature mortality among the informal caregivers [8–10]. Caregiving is, however, not always detrimental. It may also have some positive mental effects for the caregiver, especially when the burden of caregiving is low or moderate [11]. The prevalence of caregiving is mostly known to be higher among women and older people [12, 13].

Self-rated health (SRH) is one of the most widely used measures of personal health. SRH, when measured via one question, is a robust predictor of several health outcomes, such as functional ability [14, 15], returning to work after disease [16], health care utilization [17], diseases [18], morbidity [19, 20] and mortality [21–25]. It has been shown that SRH is a more reliable predictor for future health than other more objective measures [26]. SRH is often used as an outcome measure in public health-based population surveys and health service interventions because of its predictable functions. SRH is a complex predictor; possible explanations for SRH’s functioning include SRH representing an individual’s general perception of health, including biological, psychological and social dimensions. Several factors have shown associations with SRH, among them low income [27], work related factors [10], pension status [28], psychological and social factors [29].

Poor psychological health is one of the chronic health problems with highest prevalence globally, even among caregivers [4, 30]. Psychological health can be addressed in several ways; including psychological wellbeing, which is often defined as a state of being balanced both emotionally and intellectually.

One way to capture the World Health Organization concept of health proposed in 1948 is to measure a specific reference time period without health and with poor functioning [31]. This approach is most often used for tracking general population health status, but is sometimes also used in specific patient groups [32].

An informal caregiver provides care to a care recipient, who is most often an older or disabled family member, relative, friend, or neighbor. In a population study carried out in the USA nearly half of the elderly had difficulties performing daily activities without assistance or help, and this help was most often provided by informal caregivers [33].

Caregiving often means extra work and responsibility, and the caregiving role may influence everyday life in several ways. An interesting consideration that has not been fully explored is how caregiving relates to SRH, psychological wellbeing and loss of healthy time. Losses in healthy time due to caregiving situation, in particular, have not been fully explored. This study aimed to compare SRH, psychological wellbeing and recent days with poorer health in informal caregivers to non-caregivers in the general Swedish population.

## Methods

### Study population

Data from the annual repeated Swedish national public health survey “Health on equal terms” carried out from 2004 to 2013 was used for this study [34]. The national public health survey is a repeated cross-sectional postal questionnaire study that has been distributed on a yearly basis since 2004 by Statistics Sweden on behalf of the Public Health Agency of Sweden (previously the Swedish National Institute of Public Health). Each year 20,000 people aged 16 to 84 years were randomly selected from the Swedish national population registry (in 2005 to 2007, 10,000 people were selected), a total of 170,000 persons. The questionnaires were returned by post from 90,845 individuals in total, making the response rate 53.4 % (90,845/170,000). The chance of being randomly selected to the study more than 1 year was deemed negligible. There were also missing data on outcome variables ranging from 1.7 to 3.4 %. The study population consisted of slightly more women (54.8 %) than men (45.2 %). Drop-out analyses have been made on parts of the material, but not every year. The drop-out analyses were carried out by telephone interviews with non-responders, and the main result showed that non-responders do not seem to have a different response patterns than those responding to the survey.

### Predictor variables

The questionnaire contains about 85 questions, including data on the following areas:

Demographic data were collected using questions that assessed the respondent’s gender, age and educational level (categorized as compulsory school, secondary school or equivalent, or university).

Data on caregiving was assessed with the following questions: “Do you have any sick or old relative who you assist with everyday tasks, ensure or provide care for?” This question was dichotomized into either no (=0) or yes (=1).

Financial strain might occur as a problem among caregivers [35], and was therefore included in the multivariate analyses. Financial status was assessed by the question: “During the last 12 months have you had difficulties in managing your current expenses for food, rent, bills etc.?” This question was dichotomized into either no (=0) or yes (=1).

Social support is seen as an important factor for health outcomes among caregivers [36, 37]. The questionnaire contained the following question regarding perceived emotional social support: “Do you have someone you can share your innermost feelings with and feel confident in?” The following question was used to assess perceived instrumental social support: “Can you get help from someone/some people if you have practical problems or are ill?” These two questions were dichotomized into either yes (=0) or no (=1).

### Outcome variables

General SRH was assessed with the question “How do you rate your general state of health?”, and answers according to a five-point scale (very good, good, neither good nor poor, poor, very poor) and then dichotomized into either good (very good or good) or less than good (neither good nor poor, poor, very poor). In population studies, SRH is generally accepted by researchers as a valid measure for determining health status and it is common to dichotomize SRH to demonstrate persons with not-good SRH [38].

For measuring health outcomes in time the health-related quality of life measure (HRQOL) from Centers for Disease Control and Prevention (CDC) was used in brief standard (CDC HRQOL-4) [31]. Recent days with poorer physical and mental health were assessed with the following questions: “Considering your physical health, how many days, during the last 30-day period, would you say it was not good?” and “Considering your mental health, how many days, during the last 30-day period, would you say it was not good?”. Recent days without work capacity were assessed with the following questions: “How many days, during the last 30-day period, did your physical or mental health disable you from work or daily activities?”.

The total score of the General Health Questionnaire (GHQ12) was used to measure psychological wellbeing [39]. Each symptom on the scale has four responses from “better than usual” to “much less than usual.” For the purpose of this study, GHQ12 was scored using a Likert format: 0-1-2-3. The scores were summed up by adding all the items on the scale ranging from 0 to 36, where a higher score indicate lower psychological wellbeing. No standard cut-off values exist for dividing up the GHQ12 score threshold [40]; in this study the total GHQ12 score was dichotomized with a cut-off score of 12: into good psychological wellbeing (=0) and poor psychological wellbeing (=1) for the binary logistic regression analysis. The cut-off score was chosen due to a low mean score in the study population, which is often taken into account when selecting cut-off for the GHQ12 measurement [41, 42].

Information about long-term illness was collected through the question: “Do you have any long-term illness, problems following an accident, any disability or other long-term health problem?” Replies were phrased “no” (=0) or “yes” (=1).

### Analysis

The data was analyzed using chi-square and Mann–Whitney U test and differences in means were performed with independent samples T-tests. We used binary logistic regression models to analyze associations between caregiving, gender, age, education level, financial status, social support, long-term illness and SRH or psychological wellbeing. All tests were two-sided, a p-value ≤ 0.05 was considered statistically significant. HRQOL is measured in days, which is a count variable; therefore, negative binomial regression models with 95 % confidence intervals (CIs) were used to analyze the association between caregiving status, gender, age, education level, financial status, social support, long-term illness and recent days with poor health or without work capacity. The negative binomial model produced an incident rate ratio (IRR) for the predictor (IRR >1 indicates that the factor is associated with more days).

To investigate the associations between caregiving and health outcomes we chose to investigate sets of variables in stepwise multivariate regression analyses. The sets of variables included demographic, socioeconomic, social support and health-related factors successively to the multiple regression analyses. This approach has been used previously and enables to study the change in the impact of the caregiver status when adjusting for other variables. In the last step in the regression analyses the models also was adjusted for year of data collection. Statistical Package for the Social Sciences (SPSS)® version 19 (IBM SPSS Statistics for Windows, Armonk, NY: IBM Corp Chicago, IL, U.S.A.) was used for all statistical analyses.

### Ethical consideration

The research ethical committee at the Swedish National Board of Health and Welfare approved of the Swedish National Public Health Survey (8 December 2003). This present study was further approved by the regional ethical committee in Uppsala (11 November 2013).

## Results

The average age of the study population was 50.5 years and consisted of slightly more women than men. Compulsory school was the most common education level completed. The distribution of demographics and key variables in the study population are shown in Table 1.

**Table 1:** Distribution of characteristics among caregivers and none-caregivers

		Caregivers (N = 9343)	Non-caregivers (N = 76112)	Total
Sex	Male	41.0**	46.1**	45.5
	Female	59.0**	53.9**	54.5
Age	Mean (SD)	54.5 (14.8)**	49.2 (17.8)**	49.8 (17.5)
Education	Compulsory school	47.0**	43.5**	43.9
	Secondary school or equal	32.6**	34.3**	34.1
	University	20.4**	22.2**	22.0
Financial status	No problems	83.4**	85.7**	85.4
	Have problems	16.6**	14.3**	14.6
Emotional social support	No	12.7**	10.5**	10.7
	Yes	87.3**	89.5**	89.23
Instrumental social support	No	7.7**	4.5**	4.9
	Yes	92.3**	95.5**	95.1
Long-term illness	No	57.1**	63.6**	62.8
	Yes	42.9**	36.4**	37.2
Self-rated health	Poor or very poor	7.3**	5.8**	5.9
	Neither good nor poor	27.3**	22.5**	23.0
	Good or very good	65.5**	71.7**	71.1
Amount of recent days with poor health or without work capacity	Days with poor physical health, mean (SD)	7.29 (9.4)**	6.41 (9.4)**	6.68 (9,6)
	Days with poor mental health, mean (SD)	6.25 (9.0)**	5.27 (8.3)**	5.46 (8.5)
	Days without work capacity, mean (SD)	4.76 (8.9)**	4.12 (8.4)**	4.36 (8.6)
Psychological wellbeing	GHQ12^a^ MD, Mean (SD)	9, 10.1 (5.3)**	8, 9.5 (4.8)**	8, 9.5 (4.9)
	Good psychological wellbeing^b^	78.2**	82.4**	81.9
	Poor psychological wellbeing^b^	21.8**	17.6**	18.1

Figures as percentages if not stated otherwise. Pearson Chi-Square test was used for distributions and Mann–Whitney U test was used for median

**P ≤ 0.01

^a^General Health Questionnaire (GHQ12) in index-form, ranging from 0 to 36, where a higher score indicate lower psychological wellbeing

^b^GHQ12 was dichotomized, with cut-off score: 12, into good psychological wellbeing and poor psychological wellbeing

### Caregiving

There were 9,343 caregivers (10.9 %) in the study population. Among caregivers there were slightly more women than men (*p* ≤ 0.01). See Table 1.

### SRH, psychological wellbeing and recent days with poor health

A majority of the caregivers (65.5 %) and non-caregivers (71.7 %) reported good or very good general SRH. Less than good SRH was reported by 34.6 % of the caregivers and 28.3 % of the non-caregivers. The difference between caregivers and non-caregivers SRH was significant (*p* ≤ 0.01).

Caregivers reported lower psychological wellbeing, with median GHQ scores of 9 (IQR 6–12) compared to median 8 (IQR 6–12) in non-caregivers; the difference was significant (*p* ≤ 0.01). In distribution, 21.8 % of caregivers reported poor psychological wellbeing compared to 17.6 % of non-caregivers (*p* ≤ 0.01).

On average, caregivers had more days with poor physical health in the past month than non-caregivers: the amount of days with poorer physical health was on average 7.4 (SD = 9.89) among caregivers and 6.4 (SD = 9.43) among non-caregivers, conditions; t(83492) = 8.31, p ≤ 0.001. The average amount of days in the past month with poorer mental health among caregivers was 6.3 (SD = 9.04) compared to 5.3 (SD = 8.35) in non-caregivers conditions; t(83374) = 10.44, p ≤ 0.001. There was a higher amount of days without work capacity in caregivers compared to non-caregivers: 4.8 (SD = 8.88) in caregivers compared to 4.1 (SD = 8.38) in non-caregivers conditions; t(83128) = 6.78, p ≤ 0.001.

### Logistic and negative binomial regression models

Less than good SRH, caregiving, gender, age, education level, financial status, social support and long-term illness were tested using a binary logistic regression (Table 2). The significant differences between caregivers and non-caregivers in SRH in the univariate analyses remained significant, also in the adjusted logistic regression models.

**Table 2:** Results of logistic regression models of factors explaining less than good self-rated health

		Crude	Model 1	Model 2	Model 3	Model 4
		OR 95 % CI	OR 95 % CI	OR 95 % CI	OR 95 % CI	OR 95 % CI
Caregiving	Caregiver					
	No	1	1	1	1	1
	Yes	1.34** (1.28 to 1.40)	1.20** (1.14 to 1.26)	1.15** (1.09 to 1.21)	1.10** (1.05 to 1.16)	1.07* (1.01 to 1.13)
Demographic	Gender					
	Male	1	1	1	1	1
	Female	1.17** (1.13 to 1.20)	1.23** (1.19 to 1.27)	1.19** (1.16 to 1.23)	1.25** (1.21 to 1.29)	1.24** (1.20 to 1.29)
	Age	1.03** (1.03 to 1.03)	1.02** (1.02 to 1.02)	1.03** (1.03 to 1.03)	1.03** (1.03 to 1.03)	1.02** (1.02 to 1.02)
	Education level					
	University	1	1	1	1	1
	Secondary school or equal	1.36** (1.30 to 1.42)	1.47** (1.40 to 1.55)	1.39** (1.32 to 1.46)	1.38** (1.32 to 1.45)	1.38** (1.31 to 1.46)
	Compulsory school	2.47** (2.37 to 2.58)	2.19** (2.10 to 2.29)	2.06** (1.96 to 2.15)	2.02** (1.93 to 2.11)	2.02** (1.92 to 2.12)
Socioeconomic	Financial status					
	No problems	1		1	1	1
	Have problems	2.13** (2.05 to 2.22)		3.02** (2.89 to 3.15)	2.74** (2.62 to 2.87)	2.32** (2.21 to 2.44)
Social support	Emotional social support					
	Yes	1			1	1
	No	2.31** (2.22 to 2.42)			1.96** (1.86 to 2.06)	2.01** (1.89 to 2.12)
	Instrumental social support					
	Yes	1			1	1
	No	3.08** (2.89 to 3.27)			1.84** (1.71 to 1.98)	1.89** (1.74 to 2.05)
Illness	Long-term illness					
	No	1				1
	Yes	7.71** (7.46 to 7.96)				6.54** (6.31 to 6.79)

Odds ratio (OR), significant level and confidence interval (CI) for having poor SRH

SRH was dichotomized to lower than good (=1) and good or very good (=0)

*P ≤ 0.05, ** *P* ≤ 0.01

Model 1 = Cargiving + gender + age + education level, Model 2 = Model 1 + Financial status, Model 3 = Model 2 + Social support, Model 4 = Model 3 + Long-term illness + Year of data collection (not shown in table)

Caregiving had a significant association with lower psychological wellbeing on the GHQ12 scale, a finding which remained in the model adjusting for demographic, financial status, social support, and long-term illness (Table 3).

**Table 3:** Results of logistic regression models of factors explaining poor psychological wellbeing

		Crude	Model 1	Model 2	Model 3	Model 4
		OR 95 % CI	OR 95 % CI	OR 95 % CI	OR 95 % CI	OR 95 % CI
Caregiving	Caregiver					
	No	1	1	1	1	1
	Yes	1.30** (1.23 to 1.37)	1.38** (1.30 to 1.46)	1.31** (1.24 to 1.39)	1.25** (1.18 to 1.33)	1.22** (1.15 to 1.30)
Demographic	Gender					
	Male	1	1	1	1	1
	Female	1.56** (1.50 to 1.63)	1.55** (1.49 to 1.61)	1.51** (1.45 to 1.57)	1.63** (1.57 to 1.70)	1.63** (1.56 to 1.69)
	Age	0.99** (0.99 to 0.99)	0.99** (0.99 to 0.99)	0.99** (0.99 to 0.99)	0.99** (0.99 to 0.99)	0.98** (0.98 to 0.99)
	Education level					
	University	1	1	1	1	1
	Secondary school or equal	1.12** (1.06 to 1.18)	1.08** (1.03 to 1.14)	1.00 (0.95 to 1.06)	0.99 (0.94 to 1.04)	0.96 (0.91 to 1.01)
	Compulsory school	1.07* (1.01 to 1.13)	1.16** (1.10 to 1.22)	1.06* (1.01 to 1.12)	1.01 (0.96 to 1.06)	0.95 (0.90 to 1.00)
Socioeconomic	Financial status					
	No problems	1		1	1	1
	Have problems	3.58** (3.42 to 3.75)		3.02** (2.88 to 3.15)	2.65** (2.53 to 2.78)	2.41** (2.28 to 2.55)
Social support	Emotional social support					
	Yes	1			1	1
	No	3.00** (2.85 to 3.15)			2.44** (2.31 to 2.58)	2.41** (2.28 to 2.55)
	Instrumental social support					
	Yes	1			1	1
	No	3.71** (3.47 to 3.96)			2.06** (1.91 to 2.23)	2.02** (1.87 to 2.19)
Illness	Long-term illness					
	No	1				1
	Yes	2.54** (2.44 to 2.65)				2.44** (2.34 to 2.54)

General Health Questionnaire (GHQ12) was dichotomized (cut-off score: 12) into good psychological wellbeing (=0) and poor psychological wellbeing (=1)

Odds ratio (OR), significant level and confidence interval (CI) for the binary logistic regressions

*P ≤ 0.05 ** *P* ≤ 0.01

Model 1 = Cargiving + gender + age + education level, Model 2 = Model 1 + Financial status, Model 3 = Model 2 + Emotional and instrumental social support, Model 4 = Model 3 + Long-term illness + Year of data collection (not shown in table)

Caregiving is a risk factor for more days of poorer physical health in the past month in the univariate analysis (IRR = 1.14, 95 % CI: 1.11-1.16, P ≤ 0.001), as well as in the models adjusted for demographic, financial status, social support and long-term illness (adjusted IRR = 1.03, 95 % CI: 1.01-1.06, P = 0.019), see Table 4. Caregiving is also a risk factor for more recent days with poor mental health in the models adjusted for demographic, financial status, social support and long-term illness (adjusted IRR = 1.15, 95 % CI: 1.12-1.18, P ≤ 0.001), see Table 5.

**Table 4:** Incidence Rate Ratios (IRR) for number of recent days with poor physical health

		Crude	Model 1	Model 2	Model 3	Model 4
		IRR 95 % CI	IRR 95 % CI	IRR 95 % CI	IRR 95 % CI	IRR 95 % CI
Caregiving	Caregiver					
	No	1	1	1	1	1
	Yes	1.14** (1.11 to 1.16)	1.09** (1.06 to 1.11)	1.06** (1.03 to 1.08)	1.05** (1.02 to 1.07)	1.03* (1.01 to 1.06)
Demographic	Gender^a^					
	Male	1	1	1	1	1
	Female	1.24** (1.22 to 1.26)	1.29** (1.27 to 1.31)	1.27** (1.26 to 1.29)	1.29** (1.27 to 1.31)	1.27** (1.25 to 1.29)
	Age	1.01** (1.01 to 1.01)	1.01** (1.01 to 0.01)	1.01** (1.01 to 1.01)	1.01** (1.01 to 1.01)	0.99** (0.99 to 0.99)
	Education level^b^					
	University	1	1	1	1	1
	Secondary school or equal	1.21** (1.19 to 1.24)	1.27** (1.24 to 1.30)	1.23** (1.20 to 1.26)	1.22** (1.20 to 1.25)	1.17 (1.14 to 1.19)
	Compulsory school	1.65* (1.62 to 1.68)	1.61** (1.58 to 1.64)	1.55** (1.52 to 1.56)	1.54** (1.51 to 1.57)	1.43 (1.40 to 1.46)
Socioeconomic	Financial status^c^					
	No problems	1		1	1	1
	Have problems	1.50** (1.47 to 1.53)		1.62** (1.58 to 1.65)	1.54** (1.51 to 1.58)	1.33** (1.30 to 1.36)
Social support	Emotional social support^d^					
	Yes	1			1	1
	No	1.40** (1.37 to 1.43)			1.24** (1.21 to 1.27)	1.19** (1.16 to 1.22)
	Instrumental social support^d^					
	Yes	1			1	1
	No	1.62** (1.57 to 1.67)			1.29** (1.24 to 1.34)	1.24** (1.20 to 1.29)
Illness	Long-term illness^e^					
	No	1				1
	Yes	3.29** (3.24 to 3.34)				3.10** (3.05 to 3.15)

IRR, significant level and confidence interval (CI) for the risk of more recent days with poor physical health

*P ≤ 0.05, ** *P* ≤ 0.01

^a^Men = 1, women = 2

^b^Educational level was categorized as university (=1), secondary school (=2) or compulsory school (=3)

^c^No financial problems = 0, Have financial problems = 1

^d^Dichotomized to have support (=0) and no social support (=1)

^e^Dichotomized to no long-term illness (=0) and have long-term illness (=1)

Model 1 = Cargiving + gender + age + education level

Model 2 = Model 1 + Financial status

Model 3 = Model 2 + Social support

Model 4 = Model 3 + Long-term illness + Year of data collection (not shown in table)

**Table 5:** Incidence Rate Ratios (IRR) for number of recent days with poor mental health

		Crude	Model 1	Model 2	Model 3	Model 4
		IRR 95 % CI	IRR 95 % CI	IRR 95 % CI	IRR 95 % CI	IRR 95 % CI
Caregiving	Caregiver					
	No	1	1	1	1	1
	Yes	1.19** (1.16 to 1.21)	1.23** (1.20 to 1.26)	1.19** (1.16 to 1.23)	1.16** (1.13 to 1.19)	1.15** (1.12 to 1.18)
Demographic	Gender^a^					
	Male	1	1	1	1	1
	Female	1.42** (1.40 to 1.44)	1.14** (1.39 to 1.44)	1.40** (1.38 to 1.43)	1.46** (1.44 to 1.48)	1.46** (1.44 to 1.49)
	Age	0.99** (0.99 to 0.99)	0.99** (0.99 to 0.99)	0.99** (0.99 to 0.99)	0.99** (0.99 to 0.99)	0.99** (0.99 to 0.99)
	Education level^b^					
	University	1	1	1	1	1
	Secondary school or equal	1.18** (1.15 to 1.28)	1.15** (1.13 to 1.18)	1.10** (1.08 to 1.13)	1.10** (1.07 to 1.12)	1.08 (1.05 to 1.10)
	Compulsory school	1.26* (1.24 to 1.28)	1.35** (1.33 to 1.38)	1.28** (1.26 to 1.31)	1.26** (1.23 to 1.29)	1.20 (1.18 to 1.23)
Socioeconomic	Financial status^c^					
	No problems	1		1	1	1
	Have problems	2.07** (2.03 to 2.11)		1.92** (1.88 to 1.96)	1.76** (1.73 to 1.80)	1.62** (1.58 to 1.65)
Social support	Emotional social support^d^					
	Yes	1			1	1
	No	1.85** (1.81 to 1.89)			1.65** (1.61 to 1.70)	1.63** (1.59 to 1.67)
	Instrumental social support^d^					
	Yes	1			1	1
	No	2.04** (1.98 to 2.11)			1.53** (1.47 to 1.59)	1.46** (1.41 to 1.52)
Illness	Long-term illness^e^					
	No	1				1
	Yes	1.88** (1.85 to 1.91)				1.94** (1.91 to 1.97)

IRR, significant level and confidence interval (CI) for the risk of more recent days with poor mental health

* *P* ≤ 0.05, ** *P* ≤ 0.01

^a^Men = 1, women = 2

^b^Educational level was categorized as university (=1), secondary school (=2) or compulsory school (=3)

^c^No financial problems = 0, Have financial problems = 1

^d^Dichotomized to have support (=0) and no social support (=1)

^e^Dichotomized to no long-term illness (=0) and have long-term illness (=1)

Model 1 = Cargiving + gender + age + education level

Model 2 = Model 1 + Financial status

Model 3 = Model 2 + Social support

Model 4 = Model 3 + Long-term illness + Year of data collection (not shown in table)

Caregiving is a risk factor for more recent days without work capacity in the models adjusted for demographic, social support and long-term illness (adjusted IRR = 1.08, 95 % CI: 1.04-1.11, P ≤ 0.001), see Table 6. This model was tested when only including people of working age (18–65).

**Table 6:** Incidence Rate Ratios (IRR) for number of recent days without work capacity

		Crude	Model 1	Model 2	Model 3	Model 4
		IRR 95 % CI	IRR 95 % CI	IRR 95 % CI	IRR 95 % CI	IRR 95 % CI
Caregiving	Caregiver					
	No	1	1	1	1	1
	Yes	1.22** (1.19 to 1.26)	1.14** (1.11 to 1.17)	1.09** (1.06 to 1.12)	1.06** (1.03 to 1.09)	1.08** (1.04 to 1.11)
Demographic	Gender^a^					
	Male	1	1	1	1	1
	Female	1.30** (1.27 to 1.32)	1.37** (1.34 to 1.39)	1.34** (1.32 to 1.37)	1.38** (1.36 to 1.41)	1.34** (1.31 to 1.37)
	Age	1.01** (1.01 to 1.01)	1.01** (1.01 to 1.01)	1.01** (1.01 to 1.01)	1.01** (1.01 to 1.01)	0.99** (0.99 to 0.99)
	Education level^b^					
	University	1	1	1	1	1
	Secondary school or equal	1.30** (1.27 to 1.33)	1.35** (1.32 to 1.38)	1.28** (1.25 to 1.31)	1.27** (1.24 to 1.30)	1.18** (1.15 to 1.21)
	Compulsory school	1.92* (1.88 to 1.96)	1.89** (1.85 to 1.93)	1.76** (1.72 to 1.80)	1.74** (1.69 to 1.78)	1.47** (1.44 to 1.51)
Socioeconomic	Financial status^c^					
	No problems	1		1	1	1
	Have problems	2.12** (2.07 to 2.16)		2.14** (2.09 to 2.18)	1.98** (1.93 to 2.02)	1.34** (1.30 to 1.38)
Social support	Emotional social support^d^					
	Yes	1			1	1
	No	1.67** (1.63 to 1.72)			1.35** (1.31 to 1.39)	1.34** (1.30 to 1.38)
	Instrumental social support^d^					
	Yes	1			1	1
	No	2.30** (2.21 to 2.39)			1.61** (1.54 to 1.68)	1.58** (1.51 to 1.65)
Illness	Long-term illness^e^					
	No	1				1
	Yes	4.71** (4.62 to 4.79)				4.49** (4.41 to 4.58)

IRR, significant level and confidence interval (CI) for the risk of more recent days without work capacity

* *P* ≤ 0.05, ** *P* ≤ 0.01

^a^Men = 1, women = 2

^b^Educational level was categorized as university (=1), secondary school (=2) or compulsory school (=3)

^c^No financial problems = 0, Have financial problems = 1

^d^Dichotomized to have support (=0) and no social support (=1)

^e^Dichotomized to no long-term illness (=0) and have long-term illness (=1)

Model 1 = Cargiving + gender + age + education level

Model 2 = Model 1 + Financial status

Model 3 = Model 2 + Social support

Model 4 = Model 3 + Long-term illness + Year of data collection (not shown in table)

## Discussion

This study aimed to compare caregivers’ and non-caregivers’ self-rated health, psychological wellbeing, time lose to poorer health or whiteout work capacity, in a general population. Also in models adjusted for demographic, financial status, perceived social support, and long-term illness.

The main findings in this study show that caregivers reports lower health related outcomes. These results are consistent with other studies on caregiving that shows that caregiving is associated with worse health conditions [2, 4], and that caregivers report lower SRH [43]. Demographic, socioeconomic, perceived social support and long-term illness had associations with SRH, and while the effect of caregiving diminished when adjusting for these factors, it still remained statistically significant. This indicates that SRH summarizes more health outcomes and a broader concept than merely perception of illness. Most people in this study rated their health as good or very good, regardless of whether they were caregivers or not. However, from the perspective of public health the differences due to caregiving status are certainly of substantial importance. The prevalence of caregiving was significantly higher in women, which is consistent with earlier studies [12, 13].

Psychological wellbeing was negatively associated with caregiving, which indicates that caregiving has an important impact on everyday life and how people experience their life situation. These results remained significant even when the analyses included long-term illness. Zegwaard et al. found in a review study that physiological, behavioral and emotional responses such as lack of sleep, anger, irritation, headache, worry, guilt, discouragement and a poor diet are often seen among caregivers [44]. The caregiving role is also often complex and associated with stress [45], and many caregivers are often unprepared for their new role [45], therefore social support is seen as an important factor for caregivers [36]. In this study, however, the impact of being a caregiver decreased but remained statistically significant even when controlling for perceived social support, this result implies that caregivers are in need of social support, but also that it is not the only factor that is needed.

Caregivers reported more recent days with poor physical and mental health. Caregiving seems to be most strongly associated with lower mental health and this is also indicating in loss of healthy time. This implies that caregiving has negative associations with several health outcomes and that the association seems to be most evident for mental health.

Performing caregiving takes time from other activities in life, and in some cases caregivers need to take time out from work [46], which to some extent may cause problems for the caregiver in the workplace [46]. In this study, caregiving was associated with more recent days of lost work capacity.

No data was collected on the care receivers in this study, but it is common that the person in need of informal care is an older relative. The elderly population is rapidly increasing in Sweden and Europe. Thus, the number of older people requiring more care, particularly the very old (≥85 years), is expected to increase dramatically. A substantial proportion of the care is provided by unpaid family members or informal caregivers [47]. If caregiving may cause health problems among caregivers this might be an increasing problem in society when a larger proportion of the population becomes elderly and in need of formal as well as informal care. At the same time a substantial proportion of the people that represent this “resource” are suffering from lower health and poor psychological wellbeing, which may lead to less capacity to provide care for relatives.

This study provides preliminary evidence that informal caregiving is associated with worse personal perceived health, a lower level of psychological wellbeing and loss of healthy time. There is a need to further investigate this obtained association in longitudinal studies, to determine potential causality between caregiving status and health. However, this study suggests that caregiving should be considered a factor of interest when investigating personal perceived health and psychological wellbeing.

Informal caregiving is a resource for family members as well as for society and this study’s results imply is that society needs to gain more knowledge about informal caregiving and the caregiver’s possible need for support. Caregivers perform a variety of tasks and the necessities are not similar from one family to another which may complicate the design of a general intervention to this group. Maybe a new caring model, which includes the present caregiver’s own needs as a part of the care for the patient, would be beneficial to implement.

### Strengths and limitations

The strengths in this study include the community setting, the large study population and the simple randomly selected approach. The study population was very large, thus providing good power to detect differences between caregivers and non-caregivers. The response rate is reasonable according to what one expects in questionnaire studies on population level. This study also has some limitations worth noting. While we tried to perform multivariate analysis to assess the sole association of caregiving, there is a possibility that we have not included some unmeasured but important confounders. In all studies using questionnaire data, there is a risk of selection bias: in this study the risk was that more people with poorer health happened to be selected into the caregiver group, and that this affected the produced results. Being a cross-sectional design, it is not possible to determinate the temporal and cause–effect relationship between informal caregiving and health outcomes. It is, however, reasonable to assume that caregiving status may affect different health outcomes, rather than the other way around. However, more research is needed to determine what these differences in health between caregivers and non-caregivers are caused by and which factors mediate the role of being a caregiver and the associations with lower health outcomes. There is also a need to investigate which factors cause, and mediate, the role of being a caregiver to the associations with lower health outcomes. Further, report bias may exist due to the nature of self-reported data.

## Conclusion

This study suggests that caregivers have worse perceptions in self-rated health and psychological wellbeing compared with non-caregivers, indicating that the role of caregiver is adversely associated with health. This association also appears in terms of reporting days of poor health in the last month. The underlying mechanism of these associations, including the potential detrimental health effects of being a caregiver, needs to be investigated in longitudinal studies.
